# A Polyvinyl Alcohol (PVA)-Based Phantom for Prostate Cancer Detection Using Multiparametric Ultrasound: A Validation Study

**DOI:** 10.3390/bioengineering11111052

**Published:** 2024-10-22

**Authors:** Adel Jawli, Ghulam Nabi, Zhihong Huang

**Affiliations:** 1 Division of Imaging Sciences and Technology, School of Medicine, Ninewells Hospital, University of Dundee, Dundee DD1 9SY, UK; 2 Department of Clinical Radiology, Sheikh Jaber Al-Ahmad Al-Sabah Hospital, Ministry of Health, Sulaibikhat, Kuwait City 13001, Kuwait; 3 School of Science and Engineering, University of Dundee, Dundee DD1 4HN, UK

**Keywords:** multiparametric ultrasound, elastography, prostate cancer, phantom

## Abstract

Multiparametric ultrasound (mpUS) enhances prostate cancer (PCa) diagnosis by using multiple imaging modalities. Tissue-mimicking materials (TMM) phantoms, favoured over animal models for ethical and consistency reasons, were created using polyvinyl alcohol (PVA) with varying molecular weights (Mw). Methods: Four PVA samples, varying in Mw with constant concertation, were mixed with glycerol, silicon carbide (SiC), and aluminium oxide (Al_2_O_3_). Phantoms with varying depth and inclusion sizes were created and tested using shear-wave elastography (SWE). An mpUS phantom was developed to mimic prostate tissue, including isoechoic and hypoechoic inclusions and vessels. The phantom was scanned using supersonic ultrasound, strain elastography, and Doppler ultrasound. Validation was performed using radical prostatectomy data and shear-wave elastography. Results: The acoustic properties varied with enhancers like glycerol and Al_2_O_3_. Low Mw PVA samples had a speed of sound ranging from 1547.50 ± 2 to 1553.70 ± 2.2 m/s and attenuation of 0.61 ± 0.062 to 0.63 ± 0.05 dB/cm/MHz. High Mw PVA samples ranged from 1555 ± 2.82 to 1566 ± 4.5 m/s and 0.71 ± 0.02 to 0.73 ± 0.046 dB/cm/MHz. Young’s modulus ranged from 11 ± 2 to 82.3 ± 0.5 kPa across 1 to 10 freeze-thaw cycles. Inclusion size, depth, and interaction statistically affect the SWE measurements with *p*-value = 0.056327, *p*-value = 8.0039 × 10^−8^, and *p*-value = 0.057089, respectively. SWE showed isoechoic inclusions, prostate tissue, and surrounding tissue only. The Doppler velocity was measured in three different inner diameters. Conclusion: PVA mixed with enhancer materials creates an mpUS phantom with properties that mimic normal and abnormal prostate tissue, blood vessels, and soft tissue, facilitating advanced diagnostic training and validation.

## 1. Introduction

Multiparametric ultrasound (mpUS) is an advanced medical imaging procedure that uses several ultrasound techniques to provide an improved diagnostic assessment. It is commonly used in assessing prostate cancer (PCa). mpUS can be categorised into B-mode ultrasound, shear-wave elastography (SWE), Doppler ultrasound (DUS), and contrast-enhanced ultrasound (CEUS), which provide additional detection parameters such as elasticity and blood perfusion [1,2,3].

B-mode ultrasound is a medical imaging technique that utilises sound waves to create highly detailed images of internal organs. These images showcase the organs’ brightness or darkness, known as echogenicity. Echogenicity is dependent on tissue density and can help detect cancer. However, when it comes to prostate cancer, grayscale ultrasound does have some limitations. Prostate cancer typically appears iso-echogenic, meaning it has the same echogenicity as the surrounding tissue. This is due to the presence of stromal fibrosis in the prostate cancer tissue. In cases with minimal stromal fibrosis, it can result in hypoechoic PCa [4,5].

Elastography ultrasound is an effective method to determine the elastic characteristics of tissue induced by external sources. Two different methods are used in this technique: strain and shear-wave elastography. In strain elastography, the ultrasound machine sends a wave before and after probe compression. Then, a cross-correlation technique compares the results and calculates the tissue movement caused by the compression. Hard tissue has a high resistance to displacement and significant compression. Consequently, this method is subjective, as the degree of probe compression on the tissue can influence results, and it provides a qualitative evaluation of tissue properties, relying on a relative stiffness scale [6,7]. On the other hand, the SWE method is a quantitative approach that assesses the shear-wave velocity by applying an acoustic radiation force impulse (ARFI) to the tissue. This generates a shear wave; then, the machine measures the velocity of the wave in the region of interest (ROI). To estimate Young’s modulus (E), the Equation (1) relates it to the shear modulus (G) [8,9]:

(1) E=3G

With the ability to provide real-time assessment of tissue mechanical properties, a colour map is superimposed on the grayscale image. Nonetheless, SWE ultrasound has its limitations when it comes to detecting deep and heterogeneous lesions. Despite this, it still offers a high sensitivity and specificity in the detection of prostate cancer, reaching 88.6% and 97.3%, respectively [10]. Doppler and contrast-enhanced ultrasound are helpful in quantitatively assessing blood flow within the tissue. While DUS measures blood velocity, CEUS provides a dynamic perfusion measurement. In the case of neoplastic prostate, angiogenesis often takes place, leading to the formation of microvascular blood vessels that supply the tumour with oxygen [11,12]. This suggests that the metabolism of PCa is more active than Benign Prostatic Hyperplasia (BPH,) potentially resulting in higher blood velocity in microvascular vessels in PCa [13,14]. However, the resolution of Doppler ultrasound is above the resolution of microvessels in PCa, although it provides a high performance in PCa detection [15]. In contrast-enhanced ultrasound (CEUS), a specialised microbubble contrast agent is administered to enhance the visibility of microvessels. These microbubbles generate robust signals, enabling the visualisation of microvessels that are typically beyond the detection capabilities of conventional ultrasound Doppler systems. [16,17]. The CEUS procedure involves multiple measurements to differentiate between normal and abnormal tissues. However, each medical imaging technique has its own set of limitations that can impact its accuracy in different ways. Therefore, it is important to validate the quality of each imaging modality to identify and address any potential limitations. This is typically accomplished using tissue-mimicking materials (TMM) phantoms, which helps to ensure the accuracy and reliability of the imaging studies.

Tissue-mimicking materials (TMM) phantoms have traditionally been preferred over animal or cadaver models for medical imaging instrumentation training, validation, and quality assurance. This is due to ethical concerns and variations in human and animal body structure. TMMs are artificial bodies that mimic the physical properties of human tissue and are classified into natural and synthetic polymers. Polymers are comprised of numerous repeating subunits and have a structural resemblance to human tissue blocks such as collagen. For decades, agar and gelatine were recommended and used to represent human tissue.

TMMs are categorised according to the type of solvent used, including water-based, oil-based, and oil-in-hydrogel materials. Water-based materials are commonly used in ultrasound procedures because oil has been shown to reduce the phantom’s acoustic properties [18]. Agar has been a popular material-based material since 1980, as it has a higher melting point than gelatine despite both having comparable acoustical properties [19]. Although agar shares the same acoustic properties as human tissue, it falls short in representing the properties of the prostate tissue due to its limited range and tarnish over time, leading to changes in its properties. Polyvinyl alcohol (PVA), polyacrylamide (PAA), and other types of water-based material provide a wide range of acoustic and mechanical properties that match the prostate’s properties. However, PAA has a slower sound speed than PVA, and it is a toxic material that may cause peripheral neuropathy. On the other hand, PVA has several advantages, with its properties determined by factors such as its molecular weight (Mw), concentration in weight per volume (*w*/*v*), and the number of freeze-thaw cycles (FTCs). In a study conducted by Galvis-García et al. [20], the Young’s modulus of various PVA concentrations and Mw were tested, resulting in a positive effect on the Young’s modulus. Cournane et al. [21] measured the speed of sound of different PVA concentrations across multiple FTCs. Their findings showed that while the speed of sound was not affected by the number of FTCs, the elastic modulus increased with an increase in PVA concentration and FTC number. Consequently, PVA has the potential to provide a wide range of elastic modulus without changing the acoustic properties. Furthermore, it exhibits physical properties that match those of the human prostate [22]. The use of PVA has been prevalent in creating a prostate phantom [23] and a flow phantom that simulates blood vessels’ tissue [24,25,26]. Gautam et al. [23] utilised a PVA with a high molecular weight (Mw) ranging from 146,000 to 186,000 g/mol and tested its Young’s modulus until the 8th FTC for various PVA concentrations of 5%, 10%, and 15% *w*/*v*. However, the prostate phantom was only scanned for transrectal ultrasound (TRUS) biopsy validation, and cancer inclusion was not present within it. Moreover, the acoustic properties of several FTCs were not measured. Pure PVA exhibits a low backscatter, speed of sound, and attenuation coefficient, especially with low Mw [21,24,27].

Thus, this study aimed to develop a prostate phantom for multiparametric ultrasound (mpUS) and validate it using retrospective results of prostate cancer detection. The secondary objectives are to employ two types of polyvinyl alcohol (PVA) and various enhancer materials to evaluate their impact on the acoustic and mechanical properties, as well as to determine the specific PVA properties suitable for the mpUS prostate phantom, such as echogenicity variance and blood vessel inner diameter. Another secondary objective is to examine the influence of inclusion size, thickness, and depth on shear-wave elastography (SWE) detection

## 2. Materials and Methods

### 2.1. Sample Preparation

Fabricating the phantom to mimic normal and neoplastic prostate tissue requires determining the proper materials in properties, with other factors such as echogenicity, size of phantom, and depth. Therefore, it is considered to be important, at the beginning, to test the properties that play an important role in validation. This phantom will be used for B-mode, elastography, and Doppler ultrasound thus both acoustic and mechanical properties are considered.

Two types of PVA (99+% hydrolyzed) were obtained from Sigma Aldrich, St. Louis, MO, USA). The first type had an average molecular weight (Mw) of 89,000–98,000, while the second type had an average Mw of 130,000. Using these two types of PVA, four samples were prepared and divided into two groups. One group consisted of two samples made with the lower Mw PVA, and the other group consisted of two samples made with the higher Mw PVA. Each sample contained 10% (*w*/*v*) PVA, 1% (*w*/*v*) Silicon Carbide (SiC) Powder (400 Grit, from Logitech, Scotland, UK), and 0.5% (*w*/*v*) Ultra Fine Calcined Aluminum Oxide (Al_2_O_3_) Powder (0.3 micron, from Logitech, Scotland, UK). Additionally, one sample from each group included 10% (*w*/*v*) Glycerol (≥99.5% GC, 1,2,3-Propanetriol, Glycerine) from Sigma Aldrich (Germany), as shown in Table 1.

The equipment used to prepare PVA samples includes a water-bath heater (Cole Parmer BT-15) with an overhead strirrer (Digital Overhead Stirrer–DLH, VELP Scientifica, Usmate Velate, Italy). The water bath is equipped with a cover to prevent water evaporation and a hole for the stirrer. The water bath is prepared and heated to ensure that the water reaches up to 100 °C. While the water bath is heated, the TMM is prepared using de-ionized, de-gassed water that is heated with a Stuart UC152 Hot Stirrer. PVA is then mixed with SiC and Al_2_O_3_ using a Fisherbrand™ Wizard™ Infrared Vortex Mixer for 5 min at 1200 rpm. The mixture is then stirred manually with a glass rod for 5 min while being gently and slowly poured to ensure it does not stick to the rod and is mixed with the water. Each sample is then placed in the hot water bath for 2 h, and once the mixture’s temperature reaches 90 °C and the electrical stirrer is at 300 RPM, the beaker and water bath are both covered to prevent evaporation. The samples are then poured into cube silicon moulds with dimensions of 5 × 5 × 5 cm (L, W, H) and left at room temperature for 6 h to allow gases to rise. The gases are gently removed from the mould using a Lab Micro spoon, and the samples are placed in the freezer for 12 h at −22 °C and then thawed at room temperature for 6 h. During the thawing, de-ionized water was poured on top of samples inside the mould to prevent dehydration. The FTC is repeated 10 times for each sample, and after each FTC, the samples are physically tested.

### 2.2. Acoustic Properties Measurement

An echo-pulse technique described by Selfridge [28] and McClements et al. [29]. was used to measure the speed of sound and attenuation coefficient. A 5 MHz Sona-test transducer was used with a pulser/receiver (JSR Ultrasonics-DPR300, imaging Inc., Pittsford, New York, NY, USA), and both related to a Digital Oscilloscope (Keysight DSOX2004A, Malaysia), 70 MHz, 4 Channel, and 2 GS/s. The ultrasound wave and echo propagation were detected from the transducer, which was submerged in distilled, de-gassed water in the water tank at room temperature. The wave generation was set to a Pulse Repetition Frequency of 5 and Relative Gain at 50 dB. The distance between the transducer and reflector was measured before testing each sample, with the sample’s dimensions and weight. The speed of the sound of water was measured based on Equations (2) and (3):

(2) $$v_{w}=\frac{D}{\Delta t_{w}}$$

(3) $$\frac{2h}{v_{S}}+\frac{2\left(D-h\right)}{v_{\omega }}=\Delta t_{s}$$

where $v_{w}$ and $v_{S}$ are the sound velocity in water and the sample in m/s, respectively. D is the distance between the tip of the transducer and the top surface of the reflector in meters, h is the sample’s thickness in meters, $\Delta t_{w}$ and $\Delta t_{s}$ are the differences in transmission time in the water and sample in seconds, respectively, and the speed of sound of the sample was measured according to Equation (4).

(4) $$v_{s}=\frac{2h}{\Delta t_{S}-\frac{2\left(D-h\right)}{v_{\omega }}}$$

The propagation path of the ultrasound wave is from the transducer to the sample, penetrating it to the reflector, and back to the transducer. Therefore, when analysing sound propagation, it is crucial to select the appropriate time difference ($\Delta t_{S}$). The difference in time selected is the duration time between the initial signal and reflected signals, which was measured three times, and the average time was calculated.

The transmission coefficient determines the amount of ultrasound energy that can traverse different media, like tissue boundaries [30,31]. Attenuation measurements usually entail comparing the transmitted ultrasound signal to the incident signal. But when there are interfaces between different tissues or between tissue and a coupling medium, the amplitude of the transmitted signal is influenced by the transmission coefficient due to the reflection and transmission of part of the energy [32]. Hence, it’s important to account for the impact of transmission through these interfaces to obtain precise attenuation measurements. Thus, the attenuation coefficient was measured based on the transmission coefficient and the signal amplitude, as presented in Equations (5) and (6):

(5) $$T=\frac{4z_{S}z_{w}}{{\left(z_{S}+z_{w}\right)}^{2}}$$

(6) $$\alpha =20\times \log\left(\frac{T}{\frac{A^{\prime \prime }}{A^{\prime }}}\right)\times \frac{1}{2h}$$

where T is the transmission coefficient, and $z_{S}$ and $z_{w}$ are the acoustic impedance of the sample and water in Rayel, respectively. α is the attenuation coefficient in dB/cm/MHz, $A^{\prime }$ and $A^{\prime \prime }$ are the amplitude of first and second signal, respectively, and h is the sample thickness in cm. To ensure accurate measurements, we removed the reflector and stabilised the sample on the base to avoid any confusion between the signal amplitude from the base of the phantom and the reflector. Three measurements were calculated of each sample’s amplitude, and the average for precision was taken.

### 2.3. Young’s Modulus Measurement

After conducting acoustic testing on the sample, the Young’s modulus of each sample was measured three times, and an average was taken. We used an Instron single-column indentation machine to determine the elasticity of the samples through compression testing. The machine provided a curve of force (kN) and displacement (mm), which we then converted into a stress-strain curve after converting the force into N to calculate the slope based on Equation (7) in the most linear area by using MATLAB codes (MATLAB R2023b). Throughout the testing process, we utilised a 1000 N load cell from the benchtop and submerged the sample in water to minimise dehydration. During compression testing, the top platens were moved toward the sample while the lower platens remained stable. Prior to beginning the test, we achieved a pre-load of 1.5 to 2 N to ensure complete contact between the top platen and the top surface of the phantom. The compression rate was fixed for all samples at 0.5 mm/min, and we stopped the test at 2 mm [33].

(7) $$E=\frac{\sigma }{\varepsilon }=\frac{F∕A_{0}}{\Delta L∕L_{0}}$$

where E is the Young’s modulus in kPa, σ is stress in N/m^2^, and ε is strain. F is the force applied to the sample, $A_{0}$ is the original cross-sectional area of the material, ΔL is the change in length of the material, and $L_{0}$ is the original length of the material.

### 2.4. Influence of Depth and Inclusion Size on the Shear-Wave Elasticity

A series of samples were fabricated from 10% of medium molecular weight PVA and went through 5 FTCs, while adding 1% of SiC, comprising four groups, each containing four inclusions with varying dimensions (W, L, and H): 1 × 1 × 1 cm, 1 × 0.5 × 1 cm, 1 × 1 × 0.5 cm, and 0.5 × 0.5 × 0.5 cm. These inclusions were embedded at different depths within a phantom at 1 cm, 2.5 cm, 4 cm, and 5.8 cm from the top of the phantom. The samples were tested using an indentation machine in the same previous process, with an elasticity of 63.61 ± 2.7 kPa. The phantom background consisted of 3% *w*/*v* agar.

### 2.5. Statistical Analysis

The study investigated the statistical correlation between the number of FTCs and physical properties of the PVA phantom, in addition to the combined effects of depth and inclusion size on shear-wave elasticity, using the following statistical analyses: Pearson correlation, *t*-tests, and two-way ANOVA. It emphasised the significance of *p*-values and correlation coefficients in drawing meaningful conclusions from data analyses. A *p*-value less than 0.05 indicates statistical significance, while correlation coefficients close to 1 or −1 denote strong positive or negative correlations, respectively. These statistical measures guide researchers in interpreting the significance of their findings and drawing robust conclusions in fields such as medical imaging.

## 3. Results

### 3.1. Acoustic Properties

The stability of the sound speed during 10 FTCs was noticed. The higher molecular weight PVA solution consistently exhibited a faster sound propagation speed than the lower molecular weight across all freeze-thaw cycles and mediums. This indicates that the PVA’s molecular weight is a crucial determinant of the speed of sound, with higher MW resulting in greater speed. Interestingly, the presence of glycerol appeared to affect the speed of sound in both PVA solutions. The range of speed of sound of the samples 1, 2, 3, and 4 is 1547.50 ± 2, 1553.70 ± 2.2, 1555 ± 2.82, and 1566 ± 4.5 m/s, respectively.

The attenuation coefficient generally increases as the FTC number increases for all four materials. This means that the signal passing through the material loses more energy and is attenuated as the FTC number increases. Additionally, high Mw PVA samples, like group B, attenuate more of the sound energy. This variability in attenuation coefficient during FTCs is due to changes in sample shape as the FTC increases. The average attenuation coefficient for samples 1, 2, 3, and 4 is 0.63 ± 0.05, 0.61 ± 0.062, 0.71 ± 0.02, and 0.73 ± 0.046 dB/cm/MHz at 5 MHz, respectively.

Measurements were taken for all samples to examine the correlation between the number of FTCs and acoustic impedance. The findings indicate that the FTC has no discernible effect on acoustic impedance. Nevertheless, samples with a higher molecular weight (Mw) exhibit greater acoustic impedance than those with a lower Mw. The Pearson correlation analysis between the acoustic properties of the speed of sound, attenuation coefficient, acoustic impedance, and FTCs for each sample was provided and is shown in Table 2.

### 3.2. Mechanical Properties

The elastic modulus changing across the four samples is a function of FTCs. It has been noticed that the elastic modulus experiences an upward trend with rising FTCs and Mw of PVA. However, the introduction of glycerol adversely affected sample stiffness, resulting in lower Young’s modulus values than those without glycerol. Furthermore, the growth rate of stiffness gradually declined with increasing FTCs, eventually stabilising at the seventh FTC. The Pearson correlation analysis result between Young’s modulus in kPa of each sample and FTCs is shown in Table 3. The Pearson correlation analysis result between Young’s modulus in kPa of each sample and FTCs is shown in Table 4.

### 3.3. Geometric Changing

Volume, area, height, and density measurements were taken for the PVA samples at each FTC. It was observed that the volume in cm^3^ remained unchanged across all FTCs. However, it was noted that the cross-sectional area of the samples decreased as the FTCs increased, while the height of the phantom increased with each FTC. The density of all samples remained relatively constant. The average change in cross-sectional area from FTC 1 to FTC 10 for samples 1–4 was 0.99, 0.87, 0.94, and 0.84 m^2^, respectively. These results suggest that PVA with glycerol is more susceptible to changes in area than samples without glycerol.

### 3.4. Depth and Inclusion Size Effectiveness

**Figure 1:**
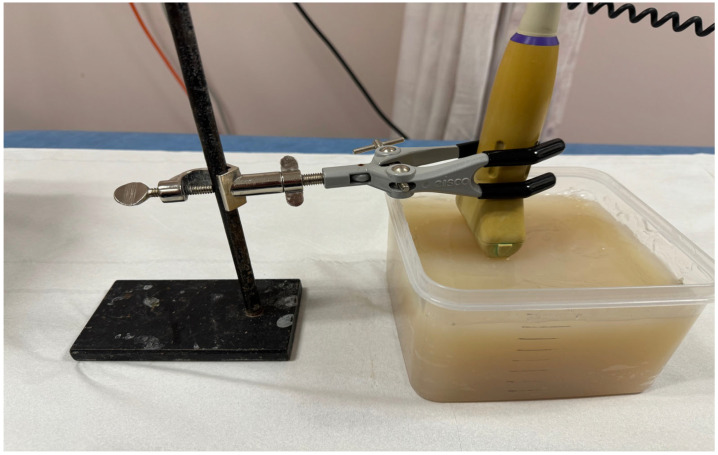
The depth phantom scanned by Axiplorer SWE-US; the transducer is held by the holder to prevent external compression.

**Figure 2:**
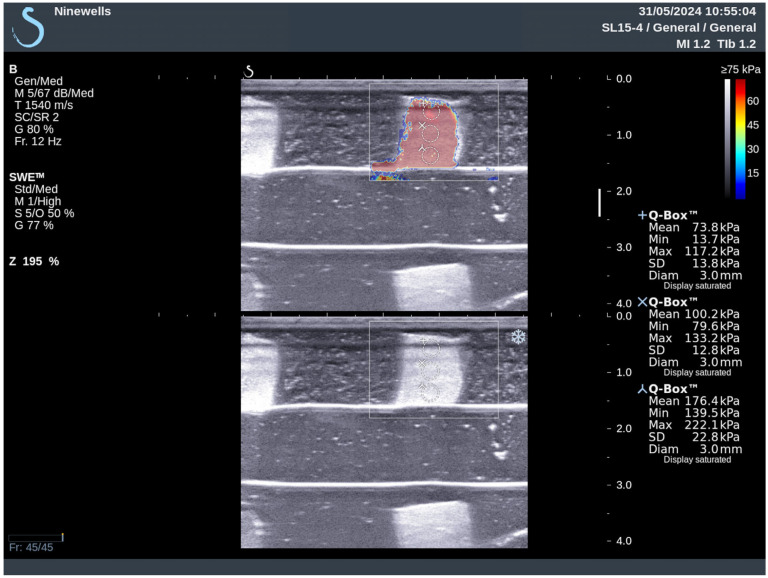
The shear-wave elasticity (kPa) for the larger inclusion of the depth phantom. The elasticity increased with the depth of inclusion.

The phantom with different sizes of PVA inclusion and different depths has been scanned by Aixplorer© (SuperSonic Imagine, Aix-en-Provence, France), as shown in Figure 1. All results using the inclusion size in different depths of the PVA are listed in Table 5. The proportion of the SWE decreased with the increase in depth, especially more than 2.5 cm. The SWE colour map in the depths of 1 cm and 2.5 cm covered the whole inclusion, while in the depth of 4 cm, the colour map partially covered the inclusion. In the depth of 5.8 cm, the SWE result was not available. The mode of SWE was changed in the depth of 5.8 cm from standard moderate mode (Std/Med) into deep penetration mode (Pen/Med), with penetration to increase the SWE penetration. Figure 2 illustrates a shear-wave ultrasound image of the multilayer phantom with multi-inclusion. It shows the colour map of the shear-wave elastography ultrasound on one inclusion in the upper layer with three measurements from the top part to the bottom part of the inclusion. It has been noticed that the shear-wave elasticity in the inclusion increased with the increase in depth. Two-way ANOVA results in the analyses of the relationship between depth, inclusion size, and elasticity indicated that the inclusion size, depth, and their interaction have a statistically significant effect on the SWE measurements based on the (*p*-value = 0.056327), (*p*-value = 8.0039 × 10^−8^), and (*p*-value = 0.057089), respectively.

## 4. Phantom Development

### 4.1. Mould Creation

**Figure 3:**
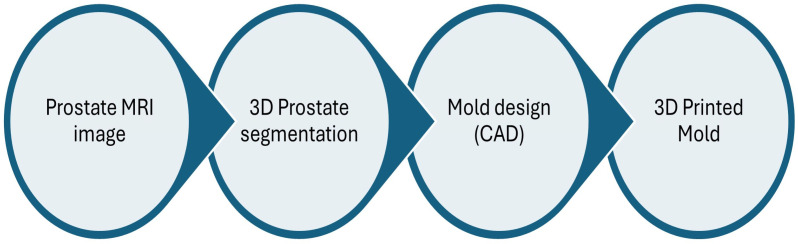
A flowchart of creating the mould of the prostate phantom.

**Figure 4:**
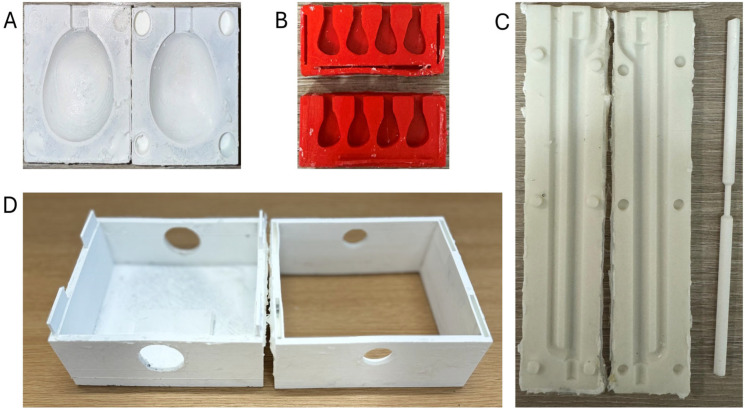
The mould of prostate phantom (**A**), inclusion phantom (**B**), blood vessels phantom (**C**), and (**D**) the external mould for the mpUS phantom.

Once the PVA sample was created and tested, the subsequent step was to develop a close prostate morphologic mould. The flowchart of this mould’s development is depicted in Figure 3. An MRI prostate image was loaded into the 3D slicer, version 4.11.20210226 software for segmentation. The segmented prostate image was inserted into SolidWorks (2023) to create a mould of the prostate. Then, a mould for inclusions, blood vessels, and the whole phantom cover was created as a solid project. A 3D printer (Ultimaker S5, Ultmaker BV, Geldermalsen, The Netherlands) was used to print the moulds. Figure 4 shows the moulds that have been printed. The first mould represents the prostate (A) and consists of two outer halves. The inner dimensions of the prostate mould are 40 mm in height, 43 mm in width, and 40 mm in depth. The mould is designed with an aperture on the upper surface, serving as a channel for inserting the PVA solution into the interior cavity. The second mould was created for inclusion (B) with the internal dimensions 14 mm, 10 mm, and 10 mm for H, W, and T (height, width, and thickness), respectively. The third mould was for the blood vessels (C), which was created based on the idea from [34] with some modification. It is a two-part mould with symmetric internal dimensions. The mould contains a cylindrical extension to allow for vessel tubing to be placed on the lumen stem, with the lumen stem’s thickness fixed at 9 mm, while the vessels measure 6 mm in diameter at the periphery and 3 mm in diameter in the middle. The mould also contains two ducts: one to allow PVA to enter the mould from the bottom and another to allow air to exit. The external mould (D) is the fourth printed mould, and it is designed to have a square shape with each side measuring 110 mm. It is divided into two separate parts. The top part has two alignment holes with a diameter of 7.5 mm for inserting valves for flow phantom connection. The bottom part has a hole with a diameter of 25 mm for the TRUS probe duct. The distance between the mid-circle of the valve hole and the TRUS hole is 30 mm.

### 4.2. Phantom Creation

Based on the properties results, both low and high Mw of PVA were utilised in all tissue materials, with the addition of Benzalkonium chloride (alkyl distribution C8-C16, 50 wt% aqueous solution, Acros organics, Geel, Belgium) at 0.5 *w*/*v* to prevent bacterial growth, as indicated in Table 6. The blood vessel tissue material was created and placed under eight FTCs, then stored in the fridge in a clean, deionized water container. Hypoechoic and isoechoic inclusions were created until they reached five and three FTCs, respectively. The material was then placed in the prostate mould and mixed with the PVA mixture to create a prostate with inclusion. To create a hole in the prostate for the blood vessels, a stick was inserted into the middle of the mould. A thin string was used to connect the inclusions and prevent flotation. The prostate phantom with inclusion was then placed in two additional FTCs. Finally, the blood vessels were inserted into the hole of the prostate, and the tissue was placed inside the external mould. PVA was poured to create the surrounding tissue and complete the FTCs. Samples were taken from the same PVA solution used to create the organ phantom to test and validate their physical properties with ultrasound results, as shown in Table 7, and these properties are compared with the properties of human tissue, as shown in Table 8.

### 4.3. Shear-Wave Elastography Scanning

**Figure 5:**
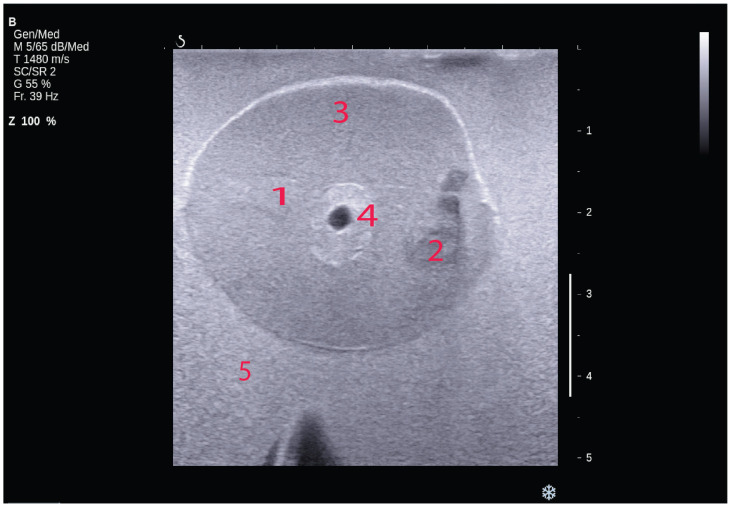
The B-mode image of the mpUS phantom with isoechoic inclusion (**1**), hypoechoic inclusion (**2**), prostate (**3**), blood vessel tissues (**4**), and surrounding tissue (**5**).

**Figure 6:**
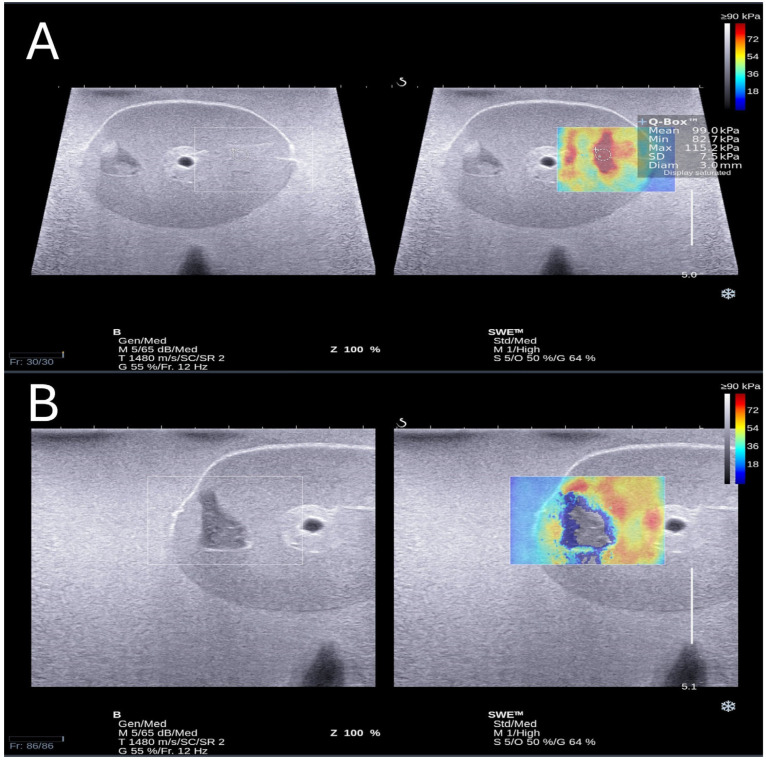
The Young’s modulus estimation of the inclusions: isoechoic inclusion (**A**) and hypoechoic (**B**) in mpUS phantom using a shear-wave elastography ultrasound.

Shear-wave elastography was used to validate the quantitative result of the phantom’s properties. The Supersonic Imaging Axiplorer (Supersonic Imagine in Aix-en-Provence, France) was used for imaging. The inclusion was prepared with different appearances and shapes to measure the Young’s modulus of the phantom. Figure 5 displays the ultrasound image of the prostate phantom, which includes two inclusions: one hypoechoic and one isoechoic, with blood vessel tissue in the middle of the prostate. The prostate had a well-defined shape, and the contrast between the prostate and surrounding tissue was evident. The isoechoic inclusion was very similar in brightness to the prostate phantom, while the hypoechoic inclusion was more heterogeneous. In Figure 6, a live shear-wave elastography ultrasound of a prostate phantom with two inclusions (hypoechoic and isoechoic) is depicted. The top image shows the colour map of the shear-wave elastography in the isoechoic inclusion, with Young’s modulus measured in kPa. However, the bottom image shows that the colour map of the shear-wave elastography in the hypoechoic inclusion could not be detected.

### 4.4. Strain Elastography Ultrasound Scanning

**Figure 7:**
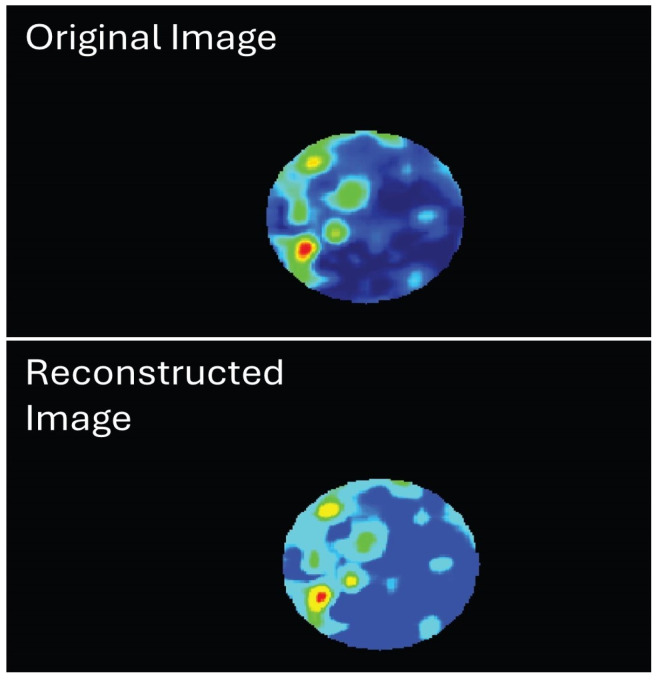
Original ROI image and reconstructed ROI image from strain elastography ultrasound.

**Figure 8:**
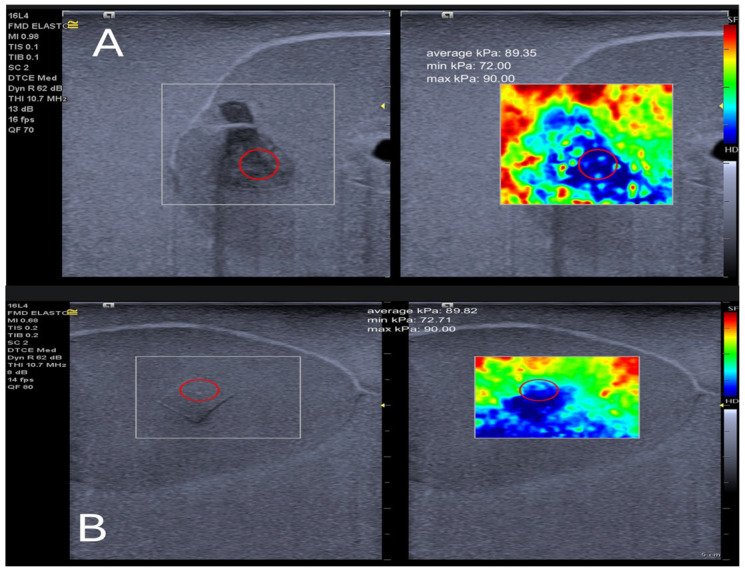
The calibrated Young’s modulus results with strain elastography ultrasound for the mpUS prostate phantom: (**A**) hypoechoic inclusion and (**B**) isoechoic inclusion., both images show the red circle indicating the selected ROI (region of interest) for calculating the elasticity in kPa.

The Siemens Acuson Juniper (Siemens Medical Solution, Mountain View, CA, USA) was utilised to perform strain elastography on the phantom. Unfortunately, the test did not yield a quantitative outcome. Strain elastography produces a colour map consisting of 255 pseudo-colour scales that range from red to blue. This colour scale is then translated into a calibrated kPa number based on the shear-wave colour scale and elasticity level [44]. The region of interest (ROI) was reconstructed using MATLAB (MATLAB R2023b). Figure 7 shows the original ROI from the strain image before recontraction (top image) and the ROI after the reconstruction method. The reconstruction process reduces pixel inhomogeneity by converting them into a similar pixel colour scale. Figure 8 displays the kPa of the strain elastography after calibration processing of the hypoechoic and isoechoic inclusion of the prostate phantom. It has been noticed that the train elastography produced the pseudo-colour scale for the heterogeneous inclusion (i.e., hypoechoic inclusion), contrasting shear-wave elastography ultrasound. In comparing Young’s modulus of phantoms using the indentation machine and shear-wave ultrasound or strain elastography, it is often observed that the indentation machine provides a higher Young’s modulus value. This observation is supported by data presented in Table 9.

### 4.5. Flow Phantom

**Figure 9:**
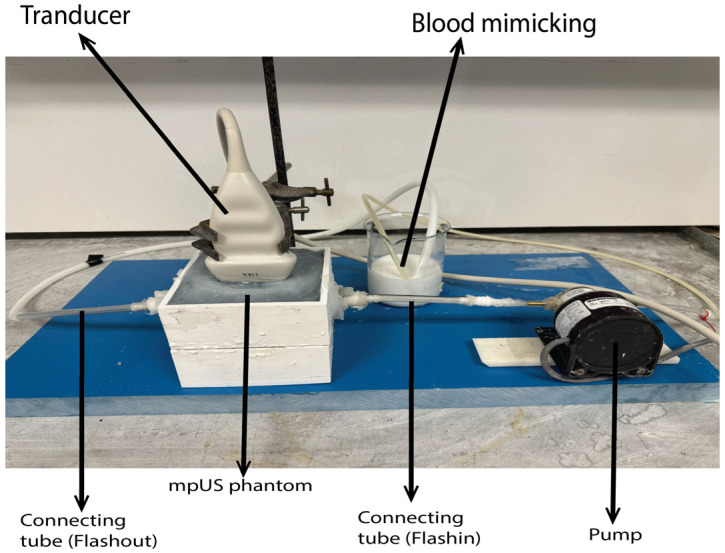
mpUS phantom used as flow phantom system, used pump, and ultrasound scanner.

**Figure 10:**
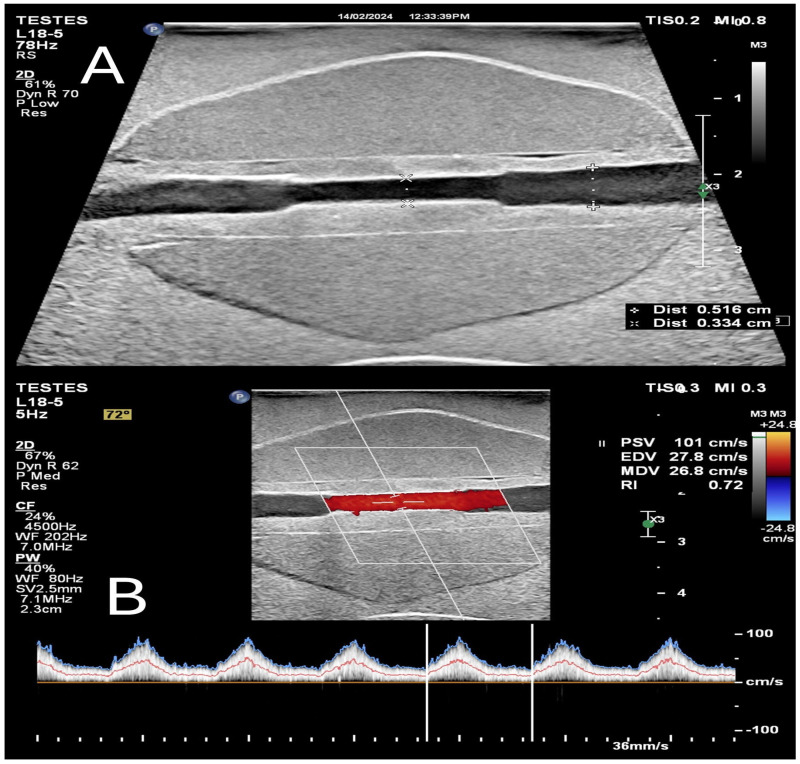
A Doppler US image of the mpUS phantom used as a flow phantom with a steady flow rate. (**A**) measured the inner diameter of the blood vessel tissue. (**B**) are the measurement of the peak systolic velocity and mean velocity. The Doppler box was used with a 72° angle to measure the velocity of the blood mimicking material inside the blood vessels (red color) at the narrowest inner diameter; doppler spectrum is shown with a blue line as the peak velocity, and a red line as the mean velocity.

An example of mpUS with a flow phantom set is shown in Figure 9. The phantom was scanned by (Epiq 5, Philips, digital ultrasound system, Bothell, WA, USA), and the blood-mimicking fluid used is commercial (Blood Mimicking Fluid Model 046, CIRS, Norfolk, VA, USA). In a flow phantom, a pump system is essential for circulating blood-mimicking materials through the phantom blood vessels, much like the heart pumps blood through the cardiovascular system. When operating a pump system, it is critical to consider its specifications, such as flow rate, based on the specific application requirements. The pump system used is from Zhou et al. [26]. Operating a computer equipped with LabVIEW 2010 (National Instruments, Austin, TX, USA) makes it feasible to produce diverse control signal waveforms. This empowers the user to design and output varied control signals. The LabVIEW setting displays the pulsatile waveform signal frequency in Hz, which represents the frequency of the human heartbeat that ranges around 60 beats per minute (bpm). To achieve this, the signal frequency is set to 1 Hz. Additionally, the signal duration, which determines the duration of each pulse in the waveform, is set to 1.4 pulses per second with an amplitude of 5 V to provide a pulse that mimics a normal artery. Upon completion of the scan, the flow phantom revealed a clear view of the blood vessels inside the prostate, with the blood-mimicking fluid present and free of air bubbles. The Doppler colour US method was used to accurately measure the peak systolic (PSV), diastolic (EDV), and mean velocity (MDV) within the widest (5 mm) and narrowest (2.5 mm) inner diameters, as depicted in Figure 10. Table 10 shows the blood velocity in the phantom in three different locations.

### 4.6. Validation with Patient Data

Shear-wave data of patients was selected retrospectively, a protocol-driven study with prior ethical approval (REC ref GTCAL11197). Thirty-five random patients, consecutive participants with clinically localised PCa who were selected and scheduled for laparoscopic radical prostatectomy, were recruited in the study between November 2013 and August 2017. The minimum, maximum, and average elasticity measured in neoplastic tissue were 74.6 kPa, 300 kPa, and 157.1 ± 80.73 kPa, respectively. In addition, the minimum, maximum, and average elasticity measured in normal prostate tissue were 5 kPa, 33.9 kPa, and 20.6 ± 5 kPa, respectively, compared to the Young’s modulus of the prostate phantom tissue to 95.85 ± 1.6 kPa, 99.35 ± 6.5 kPa, and 62.9 ± 3.5 kPa for the isoechoic and hypoechoic inclusion and prostate.

## 5. Discussion

In this study, polyvinyl alcohol (PVA) with varying molecular weights was identified as an ideal substance for creating an ultrasound phantom for multiparametric scanning. This is because the physical properties of PVA can be adjusted to resemble both diseased and normal tissue. PVA is a non-toxic synthetic polymer that contains repeating hydroxyl (-OH) groups essential for the crosslinking process. Its non-toxic nature allows it to be formed and solidified through crystallite formation from FTCs, with these crystalline structures serving as physical crosslinking points between PVA chains [45,46,47].

Acoustic properties are enhanced in the higher Mw PVA; this is due to an increase in the size of the crystallites [48]. Mawirri et al. demonstrated that polyvinyl alcohol (PVA) with a higher molecular weight (Mw) contains a greater concentration of fibres compared to PVA with a lower molecular weight. This increase in molecular weight results in a thicker PVA fibre structure. In addition, high Mw of PVA produces a phantom with high elasticity. Thus, this study’s high Mw PVA material mimics neoplastic prostate tissue. In this study, the speed of sound of the high Mw of PVA used is not very different from those in prostate cancer tissue, while the attenuation coefficient of the prostate cancer tissue is higher. The reason for using different Mw of PVA to create inclusion, although the acoustic properties of the low Mw of PVA are not too close to the prostate’s properties, is to achieve inclusion with different echogenicity and prostate cancer types. The attenuation coefficient of the prostate inclusion can be increased by either increasing the Al_2_O_3_ or increasing its particle size to 3 microns.

The stability of the speed of sound and attenuation coefficient with increasing FTCs is due to the minimal impact of the FTCs on increasing the number of hydroxyl bonds. However, the FTC number induces the crystallinity rearrangement, increasing the elasticity [49] and density, in addition to the bulk modulus, is one factor that impacts the speed of sound. A simultaneous decrease in density and increase in elasticity while increasing the FTCs may act to counterbalance each other concerning their influence on the speed of sound. The stability of the sound propagation in PVA with increasing the FTC agrees with [23], while [21] showed that the PVA sound speed increased with increasing the FTC number. This may be due to the low concentration of PVA in the sample. This can be shown by the Young’s modulus of the samples with glycerol compared to those without glycerol. The inclusion of glycerol leads to a slight decrease in Young’s modulus, mainly due to its plasticising effect on the structure of the PVA hydrogel. Glycerol interferes with the hydrogen bonding between PVA polymer chains, reducing the formation of physical cross-links. This, in turn, increases the mobility of the polymer chains, resulting in improved flexibility and reduced stiffness of the hydrogel [50].

PVA provides a wide range of elasticity while considering Mw, PVA concentration, and the number of FTCs [51,52]. This range is identical with normal and abnormal prostate tissue, as well as with blood vessels and surrounding tissue. However, this range became almost stable after the sixth FTC; this is shown in [23,51,53,54]. This is probably because the PVA mixture reaches saturation in its structure after several FTCs, and this reduces the pore’s size [49]. It is preferred to use a high Mw to simulate abnormal prostate tissue, while in this study, the isoechoic inclusion is made from low Mw to prevent any brightness or contrast difference from the prostate phantom. As discussed above, the PVA fibre of high Mw is thicker than that in low Mw, especially within a high concentration of the PVA. The result of the PVA samples and phantom parts was matching. However, the speed of sound of the prostate and vessel tissue phantom was lower and higher than expected, respectively. This is because of the slight change in the speed of sound with the change in the FTC number. This can also be shown with the attenuation coefficient in the samples and phantom parts. Attenuation coefficient measurement is not easy with the PVA phantom. It is noticed that the PVA surface after the fourth FTC does not become as flat as the first FTC. This inflates in the upper and lower surface and may impact the attenuation coefficient result.

In the depth and inclusion size impact on the shear-wave measurement, the upper part of the inclusion elasticity has a similar elasticity result to the phantom provided by the indentation machine, while the elasticity increased in the lower part of the same inclusion. This may occur because the background of the phantom was made from agar that has high elasticity. Therefore, it is noticed that in the second layer, the shear-wave elasticity is increased. In addition, inclusion with a 0.5 cm thickness might demonstrate enhanced elasticity in shear-wave elastography ultrasound due to a combination of factors. The material could have inherently greater elasticity or homogeneity in the thinner body edge effects, where closer boundaries influence shear wave propagation, which can create the impression of increased elasticity. Additionally, stress distribution in thinner bodies may lead to easier deformation under stress, resulting in a higher apparent elasticity.

In general, B-mode and shear-wave ultrasound of the prostate phantom have been validated using retrospective image data from real patients and commercial prostate phantoms. The result, overall, indicated in this study is that PVA is a suitable material when used with glycerol, SiC, and Al_2_O_3_ and is valid to simulate prostate phantom with prostate cancer. Glycerol was approved to be able to increase the speed of sound. SiC helps to create different inclusion in echogenicity by improving the backscatter. What is more, it provides a homogeneity appearance in the phantom. Al_2_O_3_ enhanced the attenuation coefficient. However, it has been shown that it can be validated with the minimum elasticity result of prostate cancer from the SWE-US scan. Consequently, this can represent prostate cancer with a low Gleason score in general. Although it is validated with commercial phantoms, our phantom is used for multiparametric ultrasound purposes. The prostate inclusion has different brightness types and different Young’s modulus. Finally, this mpUS phantom is easily scanned by a TRUS ultrasound probe and an original linear array ultrasound probe.

When comparing the Young’s modulus obtained from the compression test and shear-wave elastography, the compression test typically yields higher elasticity results. This is likely because the indentation machine measures displacement caused by a known force, while shear-wave elastography calculates Young’s modulus based on the speed of shear waves through the tissue. It is important to note that shear waves are heavily attenuated, especially at higher frequencies, leading to rapid decay. This attenuation can limit the effective propagation area, potentially making it smaller than that of the compression machine, which concentrates force on a localised area of the sample. While the results from calibrated strain elastography are similar to those from shear-wave elastography, further testing using different types of phantoms is necessary to confirm the reliability of these results. More comprehensive studies could help elucidate the relationship between shear-wave frequency, attenuation, and the resulting measurements of Young’s modulus.

In comparing shear-wave elastography images with strain elastography images, it was observed that the strain elastography ultrasound machine’s colour map effectively covered all isoechoic inclusions, unlike the shear-wave ultrasound on the Axiplorer© (SuperSonic Imagine, Aix-en-Provence, France) machine. This suggests that the Axiplorer may have limitations in detecting shear-wave elastography in heterogeneous regions, which could potentially be addressed by using a different machine. Although both inclusions in the prostate phantom have the same depth, the differences in performance between these two machines can be attributed to several factors. The new shear-wave machine likely benefits from technological advancements, providing improved sensitivity and accuracy in detecting shear waves, even in challenging regions, such as hypoechoic areas. In contrast, the older Axiplorer machine may lack the advanced algorithms and higher sensitivity required for consistent performance across different tissue types.

Flow phantoms are created with the intention of replicating important aspects of the vessels that are being studied. These aspects comprise measurements such as diameter, flow rate, blood velocity, wall motion, and flow waveform shape. It shows the pumping of blood, mimicking fluid inside the vessel’s tissue without leakage and reflection. The surrounding tissue that was made from the PVA with a low Young’s modulus allows the vessels to mimic tissues pumped with low resistance. This causes a high systolic peak velocity with a high-end diastolic peak velocity. This provides more information about changing the surrounding tissue with a stiffer material, such as agar [55,56], to figure out the result of the velocity measurement. The pumping system was close to the human heart’s blood pumping despite the pulse duration being faster than that in humans. Pulse durations lower than 1.4 pulses per second showed a low quantity of blood, mimicking fluid detection by Doppler ultrasound, which may be because of the wide inner diameter of the blood vessel tissue. The peak systolic velocity in the result is close to the normal Doppler velocity of iliac arteries, such as internal and external iliac arteries [57]. Although the diameter in the middle of the blood vessels phantom is close to the average of the prostatic artery diameter, the PSV is much higher than the Doppler velocity of the prostatic artery, especially in the BPH [58,59]. This increases due to a high pressure of the blood velocity from the side diameter to the narrow diameter. Consequently, this blood vessel phantom cannot simulate a prostatic artery.

The aim of the present study was not to manufacture a traditional phantom, the same as the commercial phantoms that are provided by CIRS (prostate training phantom), but to study how to create an inclusion isoechoic with prostate tissue and what the impact of the SWE-US on the hypoechoic lesion. In addition, it creates a phantom for multiparametric scans and different purposes, such as validation and training. The other aim is to evaluate the Doppler velocity measurement of the phantom with soft surrounding tissue instead of the usual (International Electromechanical Commission) IEC agar.

We believe that this study is a step in translational multiparametric ultrasound for prostate research. However, this study had several limitations. The ultrasound probe was not TRUS, and this may not totally impact the SWE or US result, as the phantom is not scanned in the same way as the prostate in a real patient’s scan. The vessel’s mimicking tissue is not identical to that of microvascular tissue in shape and location. Finally, the Doppler ultrasound scan is not validated due to lacking real patient data.

## 6. Conclusions

The physical properties of PVA in different Mw were measured. Based on the results, both PVA with high and low Mw provided proper fits to represent normal prostate tissue, with neoplastic prostate tissue and blood vessel tissue. Consequently, a multiparametric ultrasound phantom was fabricated. The phantom contains a prostate with different inclusions in echogenicity, vessels mimicking tissue, and surrounding tissue. The quantitative value of this phantom was obtained by shear-wave elastography ultrasound, and it was compared with the compression machine results. In addition, Doppler quantitative results were obtained. In future work, it is important to focus on enhancing the hypoechoic inclusion properties of PVA phantoms to achieve more uniform features. This enhancement could improve the accuracy of simulating soft tissue characteristics in ultrasound imaging. Additionally, conducting comparative studies between newer and older shear-wave elastography ultrasound systems is essential. These comparisons would offer insights into the limitations and advancements in elasticity detection across different generations of technology, highlighting potential areas for improvement. The creation of more realistic microvessels inside the prostate and obtaining Doppler velocity results with different surrounding tissues is recommended.

## Figures and Tables

**Table 1:** A representation of the recipes for making four samples of PVA.

	Group A		Group B	
Samples	1	2	3	4
Mw	89,000–98,000	89,000–98,000	130,000	130,000
PVA %	10	10	10	10
Glycerol %	0	10	0	10
SiC %	1	1	1	1
Al_2_O_3_ %	0.5	0.5	0.5	0.5
Water %	88.5	78.5	88.5	78.5

**Table 2:** Pearson correlation analysis between acoustic properties and FTCs for each sample, including Pearson correlation coefficient and *p*-value.

Sample	Acoustic Properties	Person Correlation Coefficient	*p*-Value
1	Speed of sound	0.32802	0.35480
2		0.51753	0.1255
3		0.63577	0.048184
4		0.59738	0.068203
1	Attenuation coefficient	0.82777	0.0031096
2		0.023607	0.94839
3		−0.50483	0.1367
4		−0.43813	0.20534
1	Acoustic impedance	−0.88632	0.00063571
2		0.19525	0.58881
3		−0.7978	0.0056834
4		−0.87383	0.00094948

**Table 3:** The elastic modulus in kPa resulted from the indentation test of the four samples of PVA.

FTC #	Sample 1	Sample 2	Sample 3	Sample 4
1	14.18 ± 1.50	11.00 ± 2.00	18.95 ± 2.00	13.39 ± 1.20
2	42.82 ± 0.20	38.82 ± 1.04	43.27 ± 0.67	41.40 ± 1.30
3	51.85 ± 1.50	50.00 ± 1.25	50.83 ± 2.00	47.50 ± 1.90
4	59.69 ± 1.86	55.80 ± 1.00	64.45 ± 1.18	54.40 ± 1.40
5	63.40 ± 4.52	60.64 ± 1.03	68.62 ± 1.02	60.80 ± 0.60
6	66.78 ± 0.30	67.92 ± 0.90	71.27 ± 2.89	64.16 ± 0.36
7	72.46 ± 0.40	71.48 ± 1.09	78.64 ± 1.47	71.87 ± 1.84
8	74.21 ± 1.18	72.23 ± 0.44	80.05 ± 1.00	75.07 ± 1.70
9	76.00 ± 3.50	75.33 ± 1.70	83.90 ± 2.06	76.12 ± 3.30
10	74.52 ± 1.70	75.64 ± 1.07	82.37 ± 0.50	77.91 ± 3.40

**Table 4:** Pearson correlation analysis between Young’s modulus, including Pearson correlation coefficient and *p*-value.

Sample	Person Correlation Coefficient	*p*-Value
1	0.88547	0.00067424
2	0.91085	0.00036009
3	0.92852	0.0001047
4	0.92793	0.00010814

**Table 5:** Shear-wave elasticity on different depths and inclusion sizes.

SWE (kPa)	Inclusion Size (High, Width, Thickness) (cm)			
Depth (cm)	1 × 1 × 1	1 × 0.5 × 1	1 × 1 × 0.5	0.5 × 0.5 × 0.5
1	116.45	70.6	104	128
2.5	134.6	135.27	224.05	179.8
4	117.6	187.27	227.65	219.2
5.8	76.93	42.67	38.65	20.2

**Table 6:** The recipes of the phantom materials.

Tissue	PVA % (Mw)	Glycerol %	SiC %	Al_2_O_3_ (0.3 µ) %	Benzalkonium Chloride	FTC #
Hypoechoic inclusion	12 (130,000)	12	0	0.5	0.5	10
Isoechoic inclusion	12 (89,000–98,000)	12	1	0.5	0.5	8
Prostate	12 (89,000–98,000)	10	1	0.5	0.5	5
Blood vessels	12 (89,000–98,000)	0	1	0	0.5	10
Soft tissue	12 (89,000–98,000)	8	15	0	0.5	3

**Table 7:** The acoustic and mechanic properties of the human tissue.

Tissue	Speed of Sound (m/s) (±SD)	Attenuation Coefficient (dB/cm/MHz) (±SD)	Young’s Modulus (kPa) (±SD)
Hypoechoic inclusion	1582 ± 1.6	0.87 ± 0.08	95.85 ± 1.6
Isoechoic inclusion	1577 ± 4	0.65 ± 0.2	99.35 ± 6.5
Prostate	1568 ± 2.8	0.7 ± 0.8	62.93 ± 3.58
Vessel’s tissue	1573 ± 5.5	0.38 ± 0.02	97.08 ± 6.6
Soft tissue	1555 ± 2.6	0.48 ± 0.15	38.1 ± 1

**Table 8:** Acoustic and mechanical properties of human tissue.

Tissue	Speed of Sound (m/s)	Attenuation Coefficient (dB/cm/MHz)	Young’s Modulus (kPa)	Refs.
Prostate cancer	1584	1.42	57–131	[10,35,36,37,38]
Prostate tissue	1561–1641	0.78	36–42	[35,36,38,39]
Vessel’s tissue	1560–1660	0.13	72–134	[40,41]
Soft tissue	1540	0.3–0.8	10	[42,43]

**Table 9:** Comparison of Young’s modulus value obtained by indentation machine, SWEUS, and calibrated strain elastography.

Phantom’s Part	Mechanical Result (kPa) ± (SD)	Strain Result (kPa) ± (SD)	SWE Result (kPa) ± (SD)
Prostate	62.93 ± 3.5	48.4 ± 6.7	47.65 ± 3.67
Isoechoic lesion	99.35 ± 6.5	86.07 ± 2.9	91.6 ± 5.5
Hypoechoic lesion	95.85 ± 1.6	81.07 ± 9.1	Not Available
Soft Tissue	38.1 ± 1	22.85 ± 4.6	20.75 ± 3.6

**Table 10:** The Doppler flow velocity was measured in different locations of the vessels and mimicking tissue.

Doppler Velocity Reading	At the Wider Inner Diameter. 5 mm	At the Beginning of Narrowing Part. 3 mm	At the Narrowing Part. 2.5 mm
PSV (cm/s)	84.3	98.6	101
EDV (cm/s)	25.9	26.8	27.8
MDV (cm/s)	23.9	24.9	26.8
RI	0.69	0.73	0.72

## Data Availability

The data presented in this study are available within this article.
